# Typological classification of attitudes toward premarital sexual behavior among Chinese undergraduates

**DOI:** 10.1186/s12889-024-20172-x

**Published:** 2024-09-27

**Authors:** Yongtao Gan, Chang Liu, Jing Deng, Jiahao Zhang

**Affiliations:** Shantou University Law College, College Road 243, Jinping District, Shantou city, 515063 Guangdong China

**Keywords:** Attitudes toward premarital sexual behavior, Typological classification, Chinese undergraduates

## Abstract

**Background:**

Premarital sexual behavior (PSB) is a controversial topic in China. However, in recent times, changes in attitudes have led to PSB being more common among college students. This study investigated the attitudes of Chinese undergraduates toward PSB to identify whether a typological classification exists among them.

**Methods:**

A total of 278 undergraduate students from two public universities in Mainland China completed a 17-item survey that included five dimensions( emotions, responsiveness, sexual health, sexual freedom, and condemnation) on attitudes toward PSB. Data were collected online from April 2023 to July 2023.

**Results:**

The PSB questionnaire demonstrated good reliability and construct validity in this study, with a Cronbach’s α of 0.759 and a KMO value of 0.769. Meanwhile, a series of models were estimated specifying one through five latent classes and three typologies on the attitude were identified: “Affective,”“Avoidant,” and “Open.” After the best fitting model was determined, multiple analysis of variance testing of different factors such as gender, year of study and where one came from were found to have significant effects on attitude complexity (*p* < .01).

**Conclusion:**

This research will contribute to the dissemination of information on PSB in China, which will be helpful in understanding relevant issues associated with PSB.

## Background

The increasing availability of and access to information on various issues, combined with changing social and cultural norms, has made premarital sexual behavior (PSB) a controversial topic in China. Although there is a longstanding tradition of advocating abstinence before marriage, recent studies have found that premarital sexual involvement among Chinese college students is becoming increasingly common [1]. Data from a 2020 survey indicated that among Chinese college students, 65.8% accepted premarital sex [2]. This phenomenon has generated a debate on the appropriate approach to address PSB.

While there is consensus that PSB carries certain risks, there is no consensus on an approach to manage it. On the one hand, some advocate for abstinence before marriage, citing traditional values and norms as the foundation for this opinion. On the other hand, some believe that sexual education and access to contraceptives should be provided to promote safe PSB. As a result, attitudes toward PSB vary.

Several studies in different communities have shown that a university lifestyle is commonly associated with risky behaviors, including sexual activities [3–5]. Therefore, all behaviors related to premarital sex are regarded as “social evils” in these societies, and contraceptive use by adolescents is not accepted [6]. Studies have demonstrated that youths’ sexual attitudes impact their sexual behavior, with more liberal attitudes leading to premarital and high-risk sexual behaviors [7]. Students’ decision to abstain from premarital sex, as suggested by motivational and post-motivational factors, including their attitude toward abstinence and social influence (such as social norms and the perceived behavior of others) on their sexual behavior and their perceived ability (self-efficacy) to remain abstinent [8].

Chinese university students’ attitudes toward premarital sex are diverse and complex. To better understand and classify such attitudes, researchers have created typological categories that categorize individuals based on their beliefs, values, and attitudes toward premarital sex. This typology provides a general framework for understanding the various attitudes toward premarital sex in China and its determining factors. The methodology follows a people-centered approach; for example, Cluster Analysis and Latent Class Analysis (LCA) can help us understand attitudes toward premarital sex for both political and educational purposes. This method allows researchers to consider different types of attitudes toward PSB for a wide range of issues identified as patterns in a population.

### The structure of forming attitudes toward PSB

Young people undergo a series of developments that result in cognitive, biological, social, mental, and physical maturity [9]. The self-image of one’s sex life (sexual esteem) and sexual needs that people develop guide their actions in this intimate sphere of life [10]. Cross-sectional surveys of adolescents and emerging adults show that pornographic consumers express more positive attitudes toward premarital sex . Several college students become addicted to sex. However, they do not consider the consequences of sex—they only want to meet their physiological needs. Individuals determine their everyday functioning, the psychological aspects of their sexuality, and the sexual activity in which they engage [11].

Several young people are comfortable with the notion of premarital romantic and/or sexual relationships, signaling a shift from an old-age, largely conservative attitude toward a more liberal and “Western” outlook [12]. Premarital sex is becoming increasingly common in romantic relationships among the young population [13]. Many college students believe premarital sex to be a commitment of their love.

Social norms regarding premarital sex have Chinese become more liberal [14]. A group study indicates that there has been a shift toward a more progressive stance on sexuality in China, characterized by an earlier initiation into sexual experiences and a broader range of sexual practices [15]. Traditional Chinese culture values conservative social norms such as responsibility, restraint, interdependence, and collectivism, suggesting that premarital sex is not endorsed, especially among single adults [16]. However, the Chinese legal system does not set legal restrictions on PSB among adults, as the traditional Chinese view on PSD is more of an informal social consensus.

In other Asian countries, such as Japan, traditional Japanese culture has historically emphasized female virginity and discouraged PSB, as part of efforts to create “good wives and wise mothers” [17]. However, public acceptance of premarital sex has been increasing in recent years [18]. Not only in Asia, but norms and values surrounding PSB have also been a subject of significant attention in other countries and regions. Traditionally, prohibitions on PSB used to play a significant role in shaping the sociosexual outlook in some groups of Americans [19].

In the United States, attitudes toward PSB are influenced by a variety of cultural, religious, and regional factors, resulting in a diverse spectrum of perspectives. Conservative religious communities, such as certain segments of conservative Christianity, often uphold traditional values and advocate for traditional gender roles and marriage as a lifelong union between man and woman, reflecting their commitment to religious teachings and family values [20]. These communities may also struggle with issues of gender identity and religious liberty, which can influence their stance on PSB. On the contrary, more liberal segments of society, especially in regions with a history of social progressivism, tend to exhibit more open attitudes toward PSB, emphasizing individual autonomy and personal choice. These liberal ideologies and attitudes are positively correlated with the values of self-direction and universalism, which are more accepting of diverse lifestyles and expressions of sexuality [21, 22].

The influence of religion on political attitudes is also significant, with religious individuals often leaning toward political conservatism and supporting existing social arrangements, including attitudes toward PSB [23]. However, it is crucial to acknowledge that not all religious individuals or communities share uniform views, and there is a wide range of beliefs and practices within any given religious tradition. In essence, the attitudes toward PSB in the United States are a complex interplay of cultural, religious, and individual factors, leading to a rich tapestry of perspectives that mirror the nation’s cultural and religious pluralism.

Despite religious beliefs and general social norms often prohibiting PSB, such as Islamic teachings explicitly forbidding premarital sex and viewing it as a sin punishable under Islamic law [24], an increasing trend in premarital sexual activity among university students has been observed even in some conservative Islamic countries such as Sudan [25].

Some believe that premarital sex is a natural process, while others think it is a crime [26]. Excessive indulgence in sexual impulses also leads to incorrect sexual consciousness and even sexual crime among college students [26]. Accordingly, PSB was considered prohibited [27]. PSB may lead to several health problems, such as at least 30 sexually transmitted infections (STIs), human immunodeficiency virus and acquired immune deficiency syndrome (HIV/AIDS), unwanted pregnancies (especially teenage pregnancy), unsafe abortions, emotional disturbances, baby dumping, and maternal death [28]. Studies have shown that several university students have borne these consequences [29].

### Typological approach to forming attitudes toward PSB

As the largest demographic group in Chinese higher education institutions, undergraduate students represent a key population for studying attitudes toward PSB. Research has identified several dimensions along which attitudes can be categorized, including religion; emotions; gender role adherence; and perceptions of social norms, beliefs, and personal experiences [13, 30, 31]. These dimensions help us understand the heterogeneous nature of attitudes within this population.

Several typological approaches have emerged in the study of attitudes toward PSB among Chinese undergraduate students [32–35]. These typologies provide insights into the diverse range of attitudes observed within a population. A typological classification developed in this field categorizes attitudes into four main types: traditional, ambivalent, liberal, and pragmatic [32]. The traditional type represents individuals who strongly adhere to traditional Chinese values and view PSB as undesirable and morally wrong. The ambivalent type reflects individuals with conflicting or mixed attitudes, often due to cultural values conflicting with modern influences [32, 34, 36, 37]. The liberal type represents individuals who have more progressive and open attitudes toward PSB, are influenced by Western values and are exposed to diverse perspectives [33, 37–40]. Finally, the pragmatic type represents individuals who hold practical and pragmatic attitudes, often shaped by individual experiences, such as personal relationships or societal pressures [41, 42]. While it is important to note that individuals may not fit perfectly into any particular typology, this provides a useful framework for understanding varying perspectives.

In summary, these typological classifications provide a valuable framework for understanding the diverse attitudes of Chinese undergraduate students toward PSB. By classifying individuals into distinct types, researchers can better analyze the factors that influence the formation of these attitudes. Factors such as family background, level of education, exposure to media, cultural values, and personal experiences all play a role in shaping an individual’s attitude type. Although other, more or fewer types may exist in other social contexts, we suggest that these four types may specifically be the case in our sample from Guangdong Province, China. Based on the literature review, the following hypotheses are proposed:

#### H1

Attitudes of Chinese undergraduates toward PSB will comprise three, four, and five latent classes.

#### H2

Demographic variables of Chinese undergraduates vary by attitude toward PSB.

## Methodology

### Sampling

This study employs convenience sampling to select undergraduate students from two public universities in Guangdong Province, China. The two public universities were selected based on their size, diversity, and reputation, ensuring a sample that could provide a comprehensive overview of the undergraduate experience in the region. Efforts were made to ensure that the sample included students from various academic disciplines, years of study, and socioeconomic backgrounds to enhance the representativeness of the findings.

Data were collected online from April 2023 to July 2023. Our study was approved by the principal of the first author’s institute. Participation was voluntary, and the participants remained anonymous. Social media platforms were used to invite as many participants as possible. After obtaining informed consent, they completed an online questionnaire. To ensure the validity of the findings, the participants had to fulfil two conditions: (1) they had to enroll in a university upon completion of their studies and (2) they could only complete one form to avoid duplication. Data from participants who had not been exposed to relevant content on premarital sexual attitudes were excluded from further analysis. Because the study focuses on analyzing individuals familiar with premarital sexual attitudes, as those unexposed could skew findings and diminish the research’s accuracy and representativeness.

### Participants

A total of 278 university students, 135 male, (48.56%) and 143 female (51.44%), participated in the study. Notably, 99 participants (35.61%) were in their second year, 83 (29.86%) in their first year, 71 (25.54%) in their first year, and 25 (8.99%) in their fourth year. The majority of 145 students (52.16%) were located in rural areas, and approximately 133 (47.84%) were located in urban areas. A total of 72 (25.90%) participants were an only child, and the majority, 206 (74.10%) had siblings.

### Data collection

Students’ attitudes toward PSB were assessed across five dimensions: emotion [12, 14], responsibility [13, 15, 17, 24, 36], sexual health [9, 11, 24, 28, 38], sexual freedom [10, 39–41], and condemnation [30, 31, 37, 38]. Table 1 presents sample items and descriptions of the five dimensions of PSB attitudes. Participants were asked to rate statements on a Likert scale from 1 (Strongly Disagree) to 5 (Strongly Agree).

**Table 1:** Description of the five dimensions and sample items exploring attitudes toward PSB

Variable (No. of items)	Description and sample items
Emotion (4)	To what extent do students believe PSB to be based on emotion. E.g., “You can have premarital sex as long as you love each other.”
Responsibility (3)	To what extent do students believe PSB is based on responsibility. E.g., “Having premarital sex is a commitment to love.”
Sexual health (3)	To what extent do students believe PSB affects health. E.g., “It is necessary to take contraceptive measures for premarital sex.”
Sexual freedom (4)	To what extent do students believe indulging in PSB is a personal freedom. E.g., “As long as two people are willing, they can have sex whether they are affectionate about each other or not.”
Condemnation (3)	To what do extent students view PSB as negative. E.g., “Premarital sex is immoral and not worth advocating.”

Note: PSB = premarital sexual behavior

The participants completed a 17-item survey that included all five dimensions. They rated their statements on a Likert scale ranging from 1 (Strongly Disagree) to 5 (Strongly Agree).

Following the classification, a more detailed study was conducted to examine the different groups and determine the proportion of each type of attitude expressed by the students. This gave the researchers a better understanding of Chinese university students’ attitudes toward premarital sex.

### Preliminary and statistical analyses

An early review of the data showed that PSB results were evenly distributed. Subsequently, a bivariate correlation analysis was performed on the key variables. Exploratory factor analyses were conducted to assess the robustness of the five PSB variables. LCA was performed using Mplus Version 8 on PSB measures. Mplus is specifically designed for complex latent variable modeling, which is essential for our study involving latent class analysis. It allows us to estimate models that are not easily handled by other software packages. One of the key advantages of Mplus is its robustness to missing data through the use of full information maximum likelihood estimation, which increases the accuracy and reliability of our results without the need for data imputation. Moreover, Mplus provides a comprehensive set of model fit indices, including Akaike Information Criterion (AIC), Bayesian Information Criterion (BIC), and Lo–Mendel–Rubin probability rate (LMR-LRT), which are critical for model selection and evaluation in latent class analysis.

Subsequently, a series of models were estimated, specifying between one and five types. Ground-to-fit studies were performed to determine the most efficient types of PSB [43]. The AIC, BIC, Akaike Bayesian Information Criterion (ABIC), and LMR-LRT were compared. Smaller AIC, BIC, and ABIC values indicated an improved model fit, whereas LMR-LRT showed that the model was more accurate than the model with fewer categories. Entropy was used to indicate how well the model was compared with other models; the entropy ranged from 0 to 1, and the larger the number, the larger the difference. Full information maximum likelihood imputation was used to replace the missing values [44]. Based on the identification of the optimal LCA model, this hypothesis was validated using independent chi-square experiments.

## Results

The PSB questionnaire’s reliability and validity have been meticulously evaluated. Additionally, the Kaiser-Meyer-Olkin (KMO) measure of sampling adequacy provided a satisfactory value of 0.785, which is indicative of adequate inter-item correlations necessary for factor analysis. This metric further supports the assertion that the PSB questionnaire possesses not only good reliability but also sound construct validity, making it a suitable instrument for the purposes of this study. The meticulous assessment of the PSB questionnaire ensures that the findings derived from its use are dependable and reflective of the underlying constructs being investigated.

A Pearson Correlation Test was also carried out in IBM SPSS26.0 to validate the effectiveness of each dimension pertaining to PSB, and the relation between the items to the scale was used, as illustrated in Table 2. The intercorrelations between the PSB dimensions, as indicated in Table 2, ranged from − 0.41 to 0.51. These coefficients provide insight into the relationships between the different dimensions of the PSB questionnaire and are essential to understand the construct validity of the instrument.

Additionally, the internal consistency of the questionnaire, as determined by Cronbach’s α, achieved a commendable score of 0.759, signifying a robust level of internal reliability. Further analysis of the individual dimensions revealed the following Cronbach’s α values: Emotion at 0.764, Responsibility at 0.621, Health at an exceptionally high 0.995, Freedom at 0.664, and Condemnation at a very strong 0.928. These scores collectively underscore the questionnaire’s consistency across its various aspects.

**Table 2:** Means, standard deviations, Cronbach’s α, and correlation coefficients (r) of the PSB dimensions (*n* = 278)

Factor^a^	1	2	3	4	5	M	SD	Cronbach’s α
Emotion	-					3.28	1.17	0.764
Responsibility	0.36**	-				2.70	1.18	0.621
Health	0.50**	0.38**	-			3.29	0.73	0.995
Freedom	0.43**	0.21**	0.51**	-		3.06	1.13	0.664
Condemnation	− 0.12*	0.06	− 0.27**	− 0.41**	-	2.44	1.10	0.928

*p < .05, **p < .01

The model fit indices for all possible solutions are presented in Table 3. In particular, the BIC and ABIC decreased as the profiles increased. The AIC decreased to 5. The Bootstrap Likelihood Ratio Test (BLRT) was statistically significant for all solutions examined, whereas there was a significant difference in the Lo-Mendell-Rubin Likelihood Ratio Test (LMRT) between the three- and four-solution models.

**Table 3:** ^**a**^. model comparison of latent profiles (*n* = 278)

Number of types	Log likelihood	AIC	BIC	ABIC	Entropy	LMRT	BLRT	Class size of type					
1	-31273.412	58666.223	62838.750	62711.874	--	--	--	278					
2	-29873.906	49779.812	59761.381	58830.540	0.935	0.0000	0.0000	22	256				
**3**	**-25330.625**	**40125.250**	**47262.118**	**47195.001**	**1.000**	**0.0000**	**0.0000**	**79**	**83**	**116**			
4	-26257.465	48570.311	50748.575	50622.303	0.941	0.0004	0.0018	22	57	83	116		
5	-27019.437	49106.874	55322.708	53214.671	0.872	0.9836	1.0000	21	57	83	116	1	

^a^Bold indicates the best fitting latent class model; AIC = Akaike Information Criterion, BIC = Bayesian Information Criterion, ABIC = Adjusted Bayesian Information Criterion, LMRT = Lo-Mendell-Rubin Likelihood Ratio Test, BLRT = Bootstrap Likelihood Ratio Test

To determine which of the five types of attitudes toward PSB had the best fit, we examined the point at which the increase in the number of profiles begin to obtain a decreasing rate of gains for the model fit. Finally, the three-section solution was considered optimal because it reached the so-called “bend” point of ABIC, where the velocity of the improved model fit begins to decline. The four-profile solution was never quantitatively distinguishable from the two-profile solution, so there was no advantage in using the four-profile solution. Thus, H1 was accepted.

### Characteristics of the three attitudes toward PSB types

**Fig. 1:**
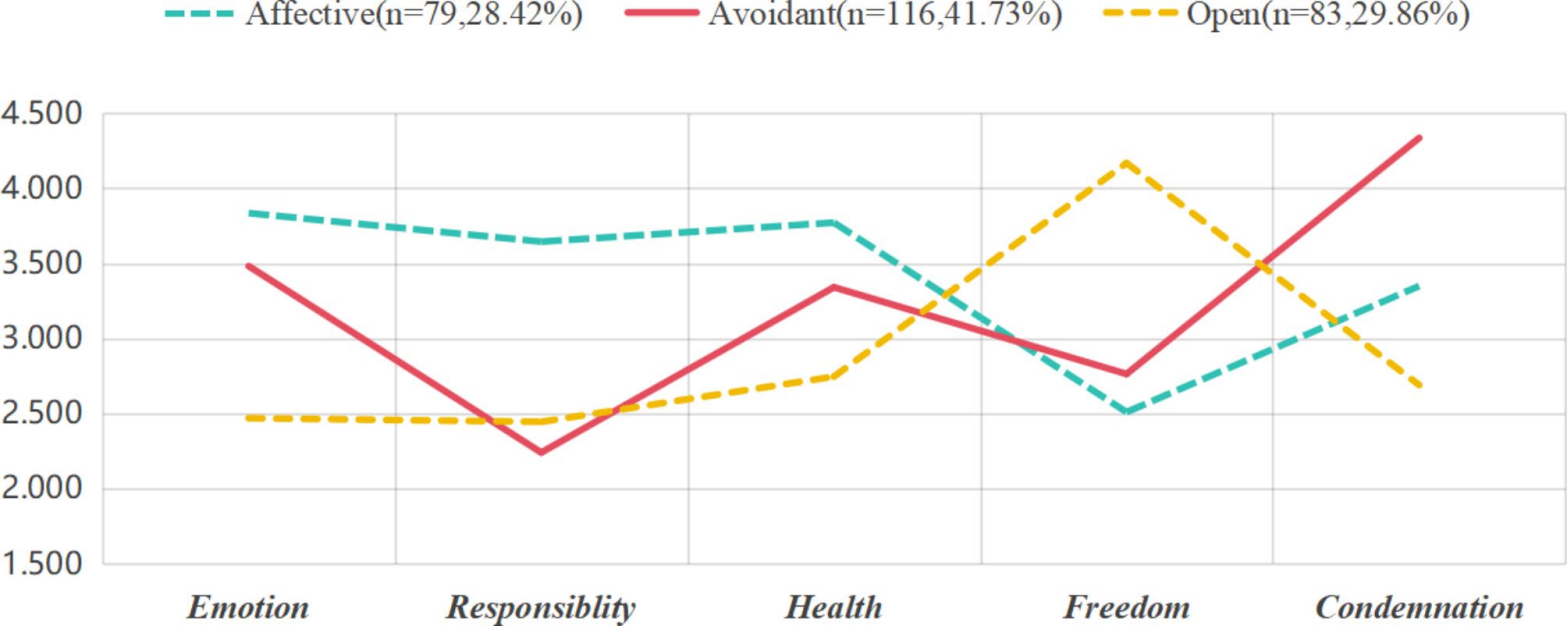
Class plot

Table 4; Fig. 1 show the class plots used to compare the three attitudes toward the PSB types: Affective, Open, and Avoidant. The numbers in Table 4 represent the mean scores for each dimension within each cluster. Higher scores indicate a more positive or open attitude toward the respective dimension of premarital sex attitudes. The dimensions are: Emotion, Responsibility, Health, Freedom, and Condemnation. The percentages in parentheses next to each cluster name indicate the proportion of the total sample that each cluster represents. For example, the Affective cluster makes up 28.42% of the sample, the Avoidant cluster 41.73%, and the Open cluster the largest portion at 29.86% .

Type 1 included 79(28.42%) of the respondents, and was named “Affective.” The Affective cluster, accounting for 28.42% of the sample size with 79 individuals, demonstrates a heightened concern for Emotion, Responsibility, and Health, scoring 3.835, 3.646, and 3.772, respectively. This suggests a more profound emotional engagement and a strong sense of duty regarding premarital sexual activity. However, this cluster shows a lower score in Freedom (2.511), indicating a more conservative view on the personal liberty aspect of premarital sex, and a moderate Condemnation score (3.350), reflecting a certain level of disapproval.

Type 2 accounted for 116(41.73%) of the respondents and was named “Avoidant”. This is the largest cluster, exhibiting lowest scores in Responsibility (2.241), which indicates a reluctance or avoidance of taking responsibility in premarital sexual contexts. Respondents’’ highest score is in Condemnation (4.336), which points to a more pronounced disapproval or negative stance toward premarital sex. The scores for Emotion (3.483) and Health (3.342) are moderate, suggesting a less intense emotional response and a cautious approach to health concerns. Respondents with avoidant attitude typology include Chinese undergraduate students who adhere to traditional values and conservative attitudes toward PSB. Different from other types, individuals falling into this typology display attitudes that do not fit neatly within any single category but rather encompass a range of beliefs and perspectives. This typology highlights the need to consider the multifaceted nature of attitudes and avoid oversimplification.

Type 3 accounted for 83(29.86%) of the respondents and was named “Open.” The Open typology exhibits lower scores across most dimensions, with the lowest scores in Condemnation (2.691), indicating a more liberal and accepting attitude toward premarital sex. Respondents’ highest score is in Freedom (4.169), reflecting a strong belief in the personal freedom to engage in premarital sexual activities. Moreover, the lowest scores were for Emotion (2.470), suggesting a more open perspective on the emotional and health-related aspects of premarital sex.

**Table 4:** Attitude-based clusters related to premarital sex attitude

Dimensions	Affective (*n* = 79, 28.42%,)	Avoidant (*n* = 116, 41.73%)	Open (*n* = 83, 29.86%)
(1) Emotion	3.835	3.483	2.470
(2) Responsibility	3.646	2.241	2.446
(3) Health	3.772	3.342	2.747
(4) Freedom	2.511	2.764	4.169
(5) Condemnation	3.350	4.336	2.691

### Demographic profile of attitudinal segments

We used a chi-square test of independence based on the demographics of the participants to identify the profiles of premarital sex-related attitudinal segments (Table 5).

Among males, the Avoidant cluster is the most prevalent (51.10%), followed by the Open (25.90%) and Affective (23.00%) clusters. For females, the distribution is more balanced, with the Affective cluster being the largest (33.60%), followed by the Open and Avoidant clusters, both at 33.60% and 32.90%, respectively. The chi-square test (χ2) shows a significant difference in the distribution of attitudes between males and females (*P* value = 0.008).

There is a significant variation in attitudes by year of study. First-year students are most likely to be in the Open cluster (42.20%), while third-year students are predominantly in the Avoidant cluster (54.90%). Most fourth-year students are represented in the Affective cluster, showing the highest percentage (48.00%). Moreover, there is a notable association between sexual experience and attitudes, with those who have had sexual experience predominantly in the Open cluster, suggesting a more liberal stance. The level of acceptance of sex education also significantly impacts attitudes. Individuals who accept sex education very well are more likely to be in the Affective cluster, while those who accept it poorly are more evenly distributed across the clusters.

Interestingly, there is no significant difference in attitudes based on whether the individual is an only child or not. Urban and rural origins do not significantly influence attitudes toward premarital sex, as indicated by the non-significant chi-square test results.

Overall, these classifications of attitudes toward PSB by demographic characteristics provide insights into the factors that may influence attitudes toward premarital sex, with significant associations found in sex, year at university, sexual experience, and acceptance of sex education.

**Table 5:** Classification profiles by demographic characteristics (%)

Demographics	Affective(in %)	Avoidant (in %)	Open(in %)	χ2 (*P* value)
**Sex**				
Male	23.00%	51.10%	25.90%	9.645 (0.008)
Female	33.60%	32.90%	33.60%	
***Year at university***				
First year	31.30%	26.50%	42.20%	30.065 (0.000)
Second year	19.20%	44.40%	36.40%	
Third year	31.00%	54.90%	14.10%	
Fourth year	48.00%	44.00%	8.00%	
***Are you an only child?***				
Yes	25.00%	40.30%	34.70%	1.219 (0.544)
No	29.60%	42.20%	28.20%	
***Where do you come from?***				
Urban	24.80%	46.60%	28.60%	2.768 (0.251)
Rural	31.70%	37.20%	31.00%	
***Have you ever had any sexual experience?***				
Yes	40.00%	5.70%	54.30%	11.220(0.003)
No	26.70%	33.30%	39.90%	
***To what extent are you accepting of sex education?***				
Very well	51.90%	29.60%	18.50%	17.413 (0.008)
Relatively well	23.90%	49.70%	26.40%	
Not well	28.70%	31.30%	40.00%	
Very poorly	37.50%	25.00%	37.50%	

## Discussion

Our survey results showed that the college students participating in our survey had different attitudes toward premarital sex. Three typologies were identified, which were labeled: open, avoidant, and affective. The findings demonstrate the differences in attitudes between individuals, which can lead to varying opinions on the issue. Furthermore, different factors such as gender, year of study, and “where are you from?” were found to significantly affect attitude complexities (*p* < .001).

### Characteristics of the three types of attitudes toward PSB

Nearly 1/2 of the Chinese undergraduates showed an avoidant/conservative attitude toward PSB. The proportion of students expressing positive attitudes (affective and open) was slightly higher than those expressing negative attitudes. This comparative analysis not only situates our findings within the extant academic dialogue but also illuminates the evolving complexity of attitudes toward PSB. It is through such scholarly discourse that we can advance our understanding of the intricate interplay between individual agency, societal norms, and the construction of sexual attitudes.

In the discourse of PSB attitudes, the present study introduces a nuanced classification system that warrants a scholarly discussion on its alignment with established typologies. The “Open” typology identified in our research resonates strongly with the “Liberal” category observed in the literature, underscoring the significance of acceptance and tolerance toward premarital sex [45]. Importantly, our conceptualization of the “Open” typology extends beyond, accentuating the primacy of individual freedom and a proactive stance on sexual behavior. This extension aligns with the “Pragmatic” category, wherein attitudes are molded by a confluence of personal encounters and societal expectations [46].

Those expressing an “open” attitude argued that if both partners agreed, then PSB can be acceptable. When studying premarital sexual attitudes, they attributed the openness of premarital sexual attitudes to physical and mental health problems. Premarital sex was also found to be more frequent among those who believed that oral contraceptive pills provided protection against STIs, including HIV [38]. Those who expressed a more open attitude toward PSB tended to have more liberal views and argued that it is permissible to engage in sexual intercourse prior to marriage.

It is noteworthy that nearly 1/2 of the respondents in our sample had a negative or even condemnatory attitude toward premarital behavior. Our identified “Avoidant” typology finds its parallel in the “Traditional” category, marked by an adherence to conventional values and a conservative or dismissive stance on premarital sexual activities [47]. The negative (avoidant, conservative) attitude of Chinese undergraduates concerning PSB is consistent with traditional practices and beliefs which state that marriage should happen before sexual intercourse [30–32]. They view premarital sex as morally wrong, valuing chastity and family honor [48, 49]. These individuals may fear social judgment, losses, and damage to their family reputations [50]. These factors demonstrate the significance of societal expectations and their impact on individual perspectives and decision-making processes [50, 51].

Intriguingly, the “Avoidant” typology in our study encapsulates elements of the “Ambivalent” category as well, reflecting the internal struggle and equivocality in attitudes attributed to the dynamic interplay between entrenched cultural norms and evolving societal views [44].

Conversely, the “Affective” typology in our research pivots on the emotional underpinnings and the sense of responsibility inherent in sexual conduct, diverging from the “Ambivalent” category’s emphasis on the fluctuating and conflicted nature of attitudes [45]. Our “Affective” typology transcends this binary, offering a holistic perspective that weaves together emotional depth, a sense of duty, and health consciousness, potentially pointing toward an emerging paradigm in the typological landscape of attitudes toward premarital sex.

“Affective” individuals believe that sex should be an expression of love rather than the sole means of satisfying desires. They emphasize that through emotional connection and mutual understanding, sexual experiences can be enriched and meaningful, while holding the viewpoint that sexual activity should not be a mere individual act but should account for the associated responsibilities [36]. They acknowledge the potential consequences of engaging in sex at both psychological and physical levels [34, 52]. They view sexual activity as a domain that should be approached from the perspective of health consciousness and safety precautions [31, 53]. They endorse the importance of sex education, including knowledge about contraception and the prevention of sexually-transmitted infections [28, 48]. These findings are consistent with previous research that supports the significance of affective attitudes toward premarital sex [31, 53]. Numerous studies have explored the attitudes of students and young adults, consistently revealing that individuals who prioritize love, responsibility, and sexual health are more inclined to adopt an affective attitude toward premarital sex rather than simply pursuing personal gratification [32, 52–56].

We grouped college students according to their attitudes toward premarital sex, and then analyzed their attitudes, so as to better understand the views of Chinese college students on premarital sex, and thus provide corresponding auxiliary measures to deal with their attitudes toward the same. The findings underscore the complex interplay between cultural norms, personal beliefs, and societal expectations in shaping individuals’ attitudes and behaviors. Chinese undergraduates were affected by traditional Chinese values concerning chastity and the cultural emphasis on virginity.

### The demographic difference among the three classes of attitude toward PSB

Our results show that, among college students’ attitudes toward PSB, men with conservative attitudes outnumbered women, indicating that gender differences should be considered in future behavioral changes. Specifically, our research shows that women are more likely to have premarital sex than men. Different from prior studies [57], is our finding that attitudes toward premarital sex are more negative in males than females. Therefore, more attention should be paid to gender differences in sex education.

#### Gender differences

This study found differences in attitudes toward PSB between sexes. Female participants were more likely to accept the idea of premarital sex than male participants. Additionally, it is essential to highlight the prominent role of females in this affective attitude toward premarital sex. Women often place greater emphasis on love, responsibility, and sexual health because of their heightened sensitivity toward physical and psychological ramifications [11]. Consequently, women play a crucial role in shaping the stance and behaviors surrounding premarital sexual activity within this affective attitude, which could be attributed to the traditional gender roles and social expectations in China that influence attitudes toward premarital sex [53]. Moreover, some studies found that female participants were more likely to have ambivalent attitudes toward premarital sex [58]. This could be interpreted as a result of the traditional values of abstinence before marriage, which is still prevalent in Chinese culture.

#### Year of study

The results indicated that there were significant differences between the different years of study in terms of attitude complexity. Freshman students exhibited a comparatively more receptive stance compared to their senior counterparts in their final year, indicating a potential trend of attitude evolution toward increased complexity as students progress through their academic tenure. Furthermore, our analysis identified a correlation between the students’ age and their attitudes toward premarital relationships. As students mature, there is a discernible shift toward a more conservative stance on premarital sex. This trend could be interpreted as a reflection of the influence of institutional education, which appears to foster a more intricate and multifaceted perspective on this social issue [59, 60].

The results highlight the relationship between students who had sexual experiences and their inclinations toward open and accepting attitudes. This finding aligns with that of previous research that suggests that individuals who have engaged in sexual activity are more open-minded and accepting of diverse sexual behaviors and orientations [10, 39–41]. One possible explanation for this finding is that personal experiences with sexuality can lead to a greater understanding and empathy toward those with differing sexual preferences [60]. Having gone through sexual experiences themselves, these students may develop broader perspectives, recognizing the complexity and diversity of human sexuality.

Additionally, the results showed that sex education shaped students’ attitudes toward PSB. Individuals demonstrating a high level of acceptance and engagement with sex education are more likely to be classified within the Affective cluster. This categorization is indicative of a robust emotional literacy and a nuanced understanding of interpersonal relationships, which are often cultivated through comprehensive sex education programs [61, 62]. In contrast, those with a moderate level of acceptance are more likely to be found in the Avoidant cluster, suggesting that their partial understanding of sex education may contribute to a defensive or dismissive attitude toward emotional intimacy [63]. Furthermore, individuals with a low level of acceptance for sex education exhibit a more uniform distribution across various clusters, implying that a lack of exposure to sex education could lead to a diverse range of emotional and relational responses, potentially due to the absence of a unifying framework for understanding sexual health and relationships [64].

### Implications

Understanding the typological approach to among Chinese undergraduate students’ attitudes toward PSB provides valuable insights into the complexities of these attitudes. Cultural and social contexts, typological variations, and multiple influencing factors have contributed to the formation of attitudes in this population. Developing comprehensive intervention strategies requires consideration of diverse typologies and context-specific approaches.

The typological classification of attitudes toward PSB among Chinese undergraduates provides valuable insights into the range of opinions for this population. This highlights the need to recognize and respect the diversity of viewpoints regarding sexual behavior, as individuals may have different cultural, religious, or personal beliefs that shape their attitudes. Further, this suggests that traditional norms surrounding PSB are evolving among Chinese undergraduates, indicating a shift toward more individualistic and pragmatic perspectives in which personal agency and practical considerations are considered.

These findings emphasize the need for effective sexual education programs that are inclusive, age-appropriate, and provide accurate information about sexual health, consent, and relationships. It is essential to provide accurate information, promote critical thinking, and foster open dialogue that cultivates a healthy understanding of sexual relationships, consent, and responsible decision-making. By equipping students with comprehensive knowledge and promoting non-judgmental attitudes, we can foster healthier and more respectful approaches to PSB.

Moreover, it is essential for educational institutions and policymakers to recognize the role of sexual education in shaping students’ attitudes. By integrating comprehensive sex education into school curricula and addressing the cultural and societal taboos surrounding sexuality, we can contribute to a more informed and accepting society. Educators should receive adequate training and support to deliver effective sex education that addresses the diverse needs and experiences of students, thereby contributing to the development of a more knowledgeable, accepting, and responsible generation.

Typological classification allows the identification of specific groups with distinct attitudes. This knowledge can guide the development of tailored interventions and support systems that cater to each group’s unique needs. Approaches should be culturally sensitive, respectful, and inclusive to promote sexual well-being and empower individuals to make informed choices.

Ultimately, typological classification offers an opportunity to promote sexual health and well-being among Chinese undergraduate students. By acknowledging and respecting diverse attitudes toward PSB, educators, policymakers, and healthcare professionals can work together to create inclusive spaces for dialogue, provide support, and promote healthy relationships and decision making.

### Limitations

It is not easy to gather sex-related information in face-to-face interviews because of the sensitivity of the topic of premarital sex. Collecting data and guaranteeing its reliability is a difficult task. Therefore, the sample size of our study was relatively small, and there were also some deficiencies in the sample distribution, such as the small proportion of senior students. However, the main goal of this study is that it can provide some insights into the correct and healthy premarital sexual behavioral attitudes of Chinese college students.

### Future research directions

Future research should focus on exploring the intersectionality of factors such as sex, socioeconomic status, and regional differences in shaping attitudes of Chinese undergraduate students toward PSB. By examining the interplay of these factors, we can better understand the complexities and variations within different typologies, and enhance our understanding of how multiple factors interact to shape attitudes.

Longitudinal studies can provide insights into the temporal changes in the Chinese undergraduate students’ attitudes toward PSB. Understanding how these attitudes evolve over time can shed light on the influence of cultural shifts, globalization, and societal norms.

Comparative studies across cultures, particularly in other East Asian countries, can contribute to a broader understanding of attitudes toward PSB. Exploring the similarities and differences between Chinese undergraduate students and their counterparts in neighboring countries can help identify culture-specific factors that influence these attitudes.

Given the increasing use of social media platforms among Chinese undergraduate students, it is crucial to investigate the influence of social media on attitudes toward PSB. Future research should examine the impact of online communities, exposure to different ideologies, and the role of online platforms in shaping attitudes and behaviors.

## Conclusions

This study explored the attitudes of Chinese undergraduates toward PSB, and identified whether typological classifications exist among them. In addition, different factors such as gender, year of study, and living area significantly affected attitude complexities. These findings have important implications for health education institutions, the public, and the government.

Based on our findings, we recommend that health education institutions develop targeted sexual education strategies; government bodies consider group differences when formulating relevant policies; university counseling centers incorporate premarital sexual attitudes into mental health assessments; and media and social organizations adopt a more objective and diverse perspective to guide public discourse. Furthermore, higher education institutions should optimize sexual health education programs to provide personalized guidance for students. Future research could explore the underlying causes of attitude formation and their relationship with actual behavior, as well as conduct cross-cultural comparative studies. This study not only enriches the existing literature but also provides valuable references for practical work, contributing to better addressing related social issues and promoting the physical and mental health development of young adults.

## Data Availability

The datasets used and/or analysed during the current study available from the corresponding author on reasonable request.Corresponding author email-id: ganyt@foxmail.com.

## Abbreviations

ABIC: Adjusted Bayesian Information Criterion
AIC: Akaike Information Criterion
AIDS: Acquired Immune Deficiency Syndrome
BIC: Bayesian Information Criterion
BLRT: Bootstrap Likelihood Ratio Test
HIV: Human Immunodeficiency Virus
LCA: Latent Class Analysis
LMRT: Lo-Mendell-Rubin Likelihood Ratio Test
PSB: Premarital Sexual Behavior
STI: Sexually Transmitted Infections
